# YOU DON’T KNOW WHAT YOU DON’T KNOW: BLACK/LATINX CANCER SURVIVORS’ KNOWLEDGE AND USE OF SURVIVORSHIP CARE PLAN

**DOI:** 10.1093/geroni/igac059.2735

**Published:** 2022-12-20

**Authors:** Candidus Nwakasi, Cristy Romero, Kateri Sinkler, Abigail Pawlowicz

**Affiliations:** Providence College, Providence, Rhode Island, United States; Providence College, Providence, Rhode Island, United States; Providence College, Providence, Rhode Island, United States; Providence College, Providence, Rhode Island, United States

## Abstract

Compared to Whites, Black and Latinx cancer survivors and their families experience disproportionate adverse effects of cancer and cancer therapy. This is due to extreme psychosocial, physical, emotional, and financial challenges they experience, thus, highlighting racial/ethnic disparity in cancer survivorship. A cancer survivorship care plan (SCP) is important for improving cancer health and quality of life, but the effectiveness of SCP as a tool to address the disproportionately persistent poor health and quality of life of Blacks/Latinx compared to Whites deserve critical attention. This study is part of a larger study on evaluating cancer survivorship in Rhode Island. We explored knowledge and use of SCP among Blacks and Latinx cancer survivors in Rhode Island. The study employed a qualitative descriptive method. Semi-structured interviews in English and Spanish were conducted with a purposive sample of 12 cancer survivors, 8 Latinx and 3 Blacks (Mage = 62 years). Their responses were transcribed and analyzed for themes. The 3 major themes identified were: 1) invisibility of survivors; 2) understanding the needs of cancer survivors; and 3) issues of empowerment. Our findings draw attention to the need for tailored interventions targeting Black/Latinx cancer survivors. One of such interventions include designing programs to increase the accessibility of SCP to help improve quality of life of cancer survivors from disadvantaged population groups.

